# High Selectivity and Reusability of Biomass-Based Adsorbent for Chloramphenicol Removal

**DOI:** 10.3390/nano11112950

**Published:** 2021-11-03

**Authors:** Weinan Xing, Qi Liu, Jingyi Wang, Siye Xia, Li Ma, Ran Lu, Yujing Zhang, Yudong Huang, Guangyu Wu

**Affiliations:** 1 Co-Innovation Center for the Sustainable Forestry in Southern China, College of Biology and the Environment, Nanjing Forestry University, Nanjing 210037, China; liuqichem@126.com (Q.L.); wangjychem@126.com (J.W.); siyexia2021@126.com (S.X.); limanjfu@163.com (L.M.); luranNJLR@126.com (R.L.); yjzhang2018@126.com (Y.Z.); 2 National Positioning Observation Station of Hung-Tse Lake Wetland Ecosystem in Jiangsu Province, Hongze 223100, China; 3 MIIT Key Laboratory of Critical Materials Technology for New Energy Conversion and Storage, State Key Laboratory of Urban Water Resource and Environment, School of Chemistry and Chemical Engineering, Harbin Institute of Technology, Harbin 150001, China; huangyd@hit.edu.cn; 4 Jiangsu Key Laboratory of Advanced Catalytic Materials and Technology, Changzhou University, Changzhou 213164, China

**Keywords:** biomass, agricultural waste, chloramphenicol removal, adsorption

## Abstract

Recently, biomass-based materials have attracted increasing attention because of their advantages of low cost, environment-friendly and nonpollution. Herein, the feasibility of using corn stalk biomass fiber (CF) and Fe_3_O_4_ embedded chitosan (CS) as a novel biomass-based adsorbent (CFS) to remove chloramphenicol (CAPC) from aqueous solution. Structure of CFS was characterized by using X-ray diffraction (XRD), Fourier transform infrared spectroscopy (FT-IR), Brunauer–Emmett–Teller (BET), scanning electron microscopy (SEM) and zeta potential techniques. The effects of solution pH, adsorption time and ion strength on the adsorption capacity were examined. Adsorption isotherms obtained from batch experiments were better fitted by Langmuir model compared with Freundlich model, Dubinin–Radushkevich model and Temkin model. Adsorption kinetic data matched well to the pseudo-second order kinetic model. CAPC adsorption was endothermic, spontaneous, and entropy-increasing nature on CFS. In addition, the CFS could be separated by an external magnetic field, recycled, and reused without any significant loss in the adsorption capacity of CAPC. Based on these excellent performances, there is potential that CFS can be considered as a proficient and economically suitable material for the CAPC removal from the water environment.

## 1. Introduction

Chloramphenicol (CAPC, Figure S1a), as a famous antibiotic, plays an important role in poultry and aquaculture around the world to control the growth and quantity of bacteria [1,2]. Specifically, in fresh water, CAPC was reported in Kenya, Korea, China, and Spain, up to 266 ng/L. Additionally, CAPC has been also detected in soils in USA, Switzerland, Mongolia, China, and Germany, at concentrations ranging from 0.1 to 2683 ng/g. CAPC has been banned in many countries especially in the field of food-producing animals, because of its side effects, like aplastic anemia, potential carcinogenicity and genotoxicity, in human health [3]. If chloramphenicol is not treated effectively, it will eventually enter the environmental system. In recent years, chloramphenicol is often detected in environmental water of China [4,5]. Therefore, it is urgent to establish an effective method to remove chloramphenicol from wastewater.

Adsorption technology has received increasing interest due to its high efficiency, simple operation, and wide applications [6,7,8,9]. Adsorbent is the key to affect the treatment effect. In recent years, more and more attention has been paid to the development of advanced adsorbents through sustainable and environmental protection methods, such as activated carbon and biomass-based material, which can be used to reduce total dissolved solids, including organic matter, dyes, metal ions and drug molecules [10,11,12]. Recently, biomass-based materials have attracted extensive attention because of their advantages of low cost, environment-friendly and nonpollution. Biomass is most often produced and can come from various sources, such as cow and pig manure, straw, fruit skins, rice, peanuts, maise, soy, wood waste, and sludge. Moreover, the surface functionality of biomass-based material could be improved by different modification methods due to the functional groups on its surface. For example, Yuan et al. prepared a Mg-Al/CSF composite adsorbent material by using waste coconut shell fiber (CSF) as the biomass carbon precursor and used for the removal of phosphorus from aqueous solution. The adsorption mechanism was the synergistic combination of surface adsorption and chemical adsorption [10]. Ahmed et al. prepared nZVI-fBC composite material with nano-Fe^0^ in situ onto surface of biomass material and investigated the removing step of chlorinated antibiotic chloramphenicol [13]. Zhao et al. synthesized a magnetic reed biochar (MRBC) with Fe^2+^/Fe^3+^ precipitated reed stalk and reported the adsorption of florfenicol (FF). The obtained maximum adsorption capacity was around 9.29 mg/g, the results of FF adsorption showed an exothermic, nonspontaneous, and entropy-decreasing step [14]. Therefore, it is feasible to prepare biomass-based materials and apply them to the separation of drug molecules.

Due to the rapid development of agricultural production and the continuous increase in the population, agricultural waste increases by 5~10% annually. At present, China’s annual output of agricultural waste has exceeded 5 billion tons by 2020, including about 0.95–1.1 billion tons of straw. Corn stalk is a common agricultural waste (Figure S1b). It is not only an important biomass resource, but also an important renewable resource. Chitosan which originates from shells of crustaceans such as crabs, prawns, krill, and crayfish is a natural, inexpensive, and nontoxic polysaccharide with a long-chain backbone comprised of N-acetyl glucosamine, obtained by the alkaline partial deacetylation of chitin. Recently, chitosan has drawn particular attention as an effective biosorbent due to its low cost compared with activated carbon and its high contents of amino and hydroxyl functional groups showing high adsorption potential for various metal ions [15,16,17,18]. In this work, the corn stalk biomass fiber (CF) was firstly prepared with corn stalk, then the functional groups of CF were reacted with –OH and –NH_2_ groups of chitosan (CS). Fe_3_O_4_ has small particle size, low toxicity, and easy separation property; its surface can also be modified with some substances. Herein, biomass-based material (CFS) adsorbent was prepared by a step-by-step method to remove chloramphenicol (CAPC) from simulation wastewater. The adsorbent was characterized by XRD, FT-IR, BET, SEM and zeta potential techniques. The pH effect, kinetic, equilibrium isotherms, and thermodynamic parameters were also addressed. The adsorption mechanism was also supplied. The obtained results show that CFS is a proficient and environmentally friendly material in wastewater treatment containing chloramphenicol.

## 2. Materials and Methods

### 2.1. Chemicals and Reagents

Abandoned corn stalks were collected from the surrounding market and was used to produce corn stalk biomass fiber. Sodium chlorite (NaClO_2_, Sinopharm Chemical Regent Co., Ltd., Shanghai, China), NaOH (Sinopharm Chemical Regent Co., Ltd., Shanghai, China), HAc (Sinopharm Chemical Regent Co., Ltd., Shanghai, China), NaAc (Sinopharm Chemical Regent Co., Ltd., Shanghai, China), NaCl (Sinopharm Chemical Regent Co., Ltd., Shanghai, China), FeCl_3_·6H_2_O (Sinopharm Chemical Regent Co., Ltd., Shanghai, China, 99.0%), Na_3_C_6_H_5_O_7_·2H_2_O (Sinopharm Chemical Regent Co., Ltd., Shanghai, China), chitosan (CS, Sinopharm Chemical Regent Co., Ltd., Shanghai, China) and HCl (Sinopharm Chemical Regent Co., Ltd., Shanghai, China, 37%). Chloramphenicol (CAPC) was provided from Aladdin Reagent Co., Ltd. (Shanghai, China).

### 2.2. Synthesis of Adsorbent

The detailed preparation for corn stalk biomass fiber (CF) was described by Yuan et al. [10] method. Briefly, 100 g of corn stalk was dried at 60 °C for 6 h, cut into pieces, and placed in a NaOH solution (1 M, 500 mL). The solid was then stirred at 85 °C for 4 h. The materials were washed with deionized water three times and dispersed in a 150 mL solution of acidified NaClO_2_ (5%) and 5 mL of HAc at 90 °C. After 3 h, the fiber product was washed with deionized water six times and collected. The obtained corn stalk biomass fiber (CF) was dried at 60 °C for 10 h. Modification of CF with chitosan: 20.0 g of chitosan was added in 80 mL NaOH and stirred at room temperature for 5 h. Then, 4.0 g of CF was added the above solution. The product (CCF) was dried at 60 °C overnight for use. The approach for synthesizing Fe_3_O_4_ was reported by Zhu et al. [19]. Briefly, 27.0 g of FeCl_3_·6H_2_O, 30.0 g of NaAc and 9.0 g of Na_3_C_6_H_5_O_7_·2H_2_O were dissolved in ethylene glycol (450 mL) under stirring and the mixture was transferred into an autoclave (200 °C) for 10 h. The final Fe_3_O_4_ were collected, washed with ethanol and deionized water three times before drying at 100 °C for a night.

CCF and solid NaOH were placed in a flask with 300 mL deionized water and stirred at 55 °C for 6 h. The solution was cooled down to room temperature and washed three times with ethanol and three times with deionized water. Subsequently, 20.0 g Fe_3_O_4_ was dissolved in 200 mL deionized water to form a homogeneous solution and the obtained solution was slowly added the above mixture. The mixture was stirred at room 25 °C for 2 h. NaHCO_3_ solution (10 wt%) was used to neutralize the above solution. The CFS was washed by deionized water 5 times and dried at 80 °C for 24 h [20].

### 2.3. Adsorption Properties

To evaluate the influence of pH, temperature and incubation time, batch mode adsorption experiments were carried out under ambient condition. Then, 1.0 g of CAPC was dissolved in ethanol (1000 mL) to obtain the primary solution. The detailed process for testing the adsorption capacity of CFS was shown as follows: the adsorbent (30 mg) was mixed with CAPC solution (20 mL), and placed in constant temperature oscillator (WSZ-35A, Qingdao Mingbo Environmental Protection Technology Co., Ltd., Qingdao, China) at 35 °C. After a reaction time of 3 h, the adsorbent material was collected with a magnet. CAPC concentration in the supernatants was measured at 278 nm by using a UV-Vis spectrophotometer (UV-2450, Shimadzu Company, Kyoto, Japan). The adsorption capacity, *q_e_*, was calculated by the following Equation (1):

(1) $$q_{e}=\frac{\left(C_{0}-C_{e}\right)}{m}\times V$$

where *q_e_* is the adsorption capacity of CFS (mg/g), *C*_0_ is the original concentration of CAPC (mg/L) and *C_e_* is the equilibrium concentration of CAPC (mg/L) [6], *m* is the weight of CFS material (g), and *V* is the volume of the liquid (L) [20].

### 2.4. Reuse Studies

In the reuse studies, methanol/acetic acid (8:2, *v*/*v*) solution was used as desorption eluent in the desorption process. Briefly, the captured CFS was dispersed in 25 mL of methanol/acetic acid solution under ultrasonic for 1.5 h. Then, the CFS was magnetically separated from the solute, washed with distilled water for several times and dried in vacuum for the next adsorption experiment. The adsorption-desorption procedure was repeated five times to evaluate the reuse performance of the sample.

### 2.5. Characterization

FT-IR spectrum of the sample was performed with an FT-IR spectrometer (AVATAR 360, Nicole company, Brunswick, NC, United States). A minimum of 32 scans was signal-averaged with a resolution of 2 cm^−1^ in the 4000~400 cm^−1^ ranges. X-ray diffraction (XRD) analysis of the material was conducted on X-ray powder diffraction (XRD-6100, Shimadzu company, Kyoto, Japan) with a scanning rate of 5°/min. The morphology of the as-prepared adsorbent was characterized by scanning electron microscope (SEM, S-4800, Hitachi company, Tokyo, Japan). The specific surface area and the pore diameter distributions were calculated using Brunauer–Emmett–Teller (BET) nitrogen adsorption-desorption isotherms and the Barrett–Joyner–Halenda (BJH) method, respectively by using a Micromeritics TriStar II 3020 analyzer (Micromeritics Instrument Corporation, Gwinnett, GA, US) at 77 K. The zeta potential of material was tested by using a Zeta potential analyzer (Zetasizer Nano ZS90, Malvern Instruments Ltd., Worcestershire, UK).

### 2.6. Adsorption Isotherm

In this work, Langmuir isotherm model (LIM), Freundlich isotherm model (FIM), Temkin isotherm model (TIM) and Dubinin–Radushkevich isotherm model (DRIM) were used to analyze the adsorption process [21,22,23,24,25],

(2) $$\mathrm{The}\;\mathrm{LIM}\;\mathrm{equation}:\;\frac{C_{e}}{q_{e}}=\frac{C_{e}}{q_{m}}+\frac{1}{K_{L}q_{m}}$$

(3) $$\mathrm{The}\;\mathrm{FIM}\;\mathrm{equation}:\;\;\;\;\ln q_{e}=\ln K_{f\;}+\frac{1}{n}\times \ln C_{e}$$

(4) $$\mathrm{The}\;\mathrm{TIM}\;\mathrm{equation}:q_{e}=(\frac{RT}{b_{T}})\ln K_{T}+\left(\frac{RT}{b_{T}}\right)\ln C_{e}$$

(5) $$\mathrm{The}\;\mathrm{DRIM}\;\mathrm{equation}:\;\ln q_{e}=\ln q_{m}-K_{DR}\varepsilon^{2}$$

(6) $$\;\varepsilon =RT\ln\left[1+\frac{1}{C_{e}}\right]$$

where: *q_e_* is the adsorption capacity at adsorption equilibrium (mg/g). *q_m_* is maximum adsorption capacity (mg/g). *C_e_* is the equilibrium concentration of CAPC solution (mg/L). *K_L_* is the Langmuir constant (L/mg). *K_f_* is the Freundlich constant (mg^1−(1/n)^ L^1/n^ g^−1^), and 1/*n* is the parameter characterizing the energy heterogeneity of the adsorption surface. *b_T_* is the Temkin constant related to heat of adsorption (J^.^g/mol^.^mg). *K_T_* is the Temkin isotherm equilibrium binding energy constant (L/mg). *R* is the gas constant (8.314 J/mol^.^K). *T* is the absolute temperature (K). *K_DR_* is the constant related to the adsorption energy (mol^2^ J^2^). *ε* is the adsorption potential (J/mol).

In the adsorption process, *R_L_* was determined to evaluate the selectivity system of adsorbate/adsorbent. *R_L_* could be calculated as Equation (7) [26]:

(7) $$R_{L}=\frac{1}{1+K_{L}C_{0}}$$

If the *R_L_* > 1, it is an unfavorable adsorption; *R_L_* = 1, it means linear adsorption; 0 < *R_L_* < 1, it is a favorable adsorption; *R_L_* = 0, it means irreversible adsorption.

### 2.7. Adsorption Kinetics

Pseudo-first-order (PFO), pseudo-second-order (PSO) and internal diffusion kinetic (IDK) models were used to analyze and explain the different steps involved in the adsorption process. The mathematical formulas used for the models are as follows Equations (8)–(10):

(8) $$\ln\left(q_{e}-q_{t}\right)=\ln q_{e}-K_{1}t$$

(9) $$\;\frac{t}{q_{t}}=\frac{1}{K_{2}q_{e}^{2}}+\frac{t}{q_{e}}$$

(10) $$q_{t}=K_{p}t^{\frac{1}{2}}+C$$

where *q_e_* (mg/g) and *q_t_* (mg/g) are the amount of CAPC adsorbed on CFS adsorbent at equilibrium and time *t* (min), respectively. *K*_1_ (min^−1^), *K*_2_ (g mg^−1^ min^−1^) and *K_p_* (mg g^−^^1^ min^1/2^) are the rate constants of adsorption for the PFO, PSO and IDK model [10,11].

## 3. Results and Discussion

### 3.1. Composition and Structure of the CFS Adsorbent

**Figure 1:**
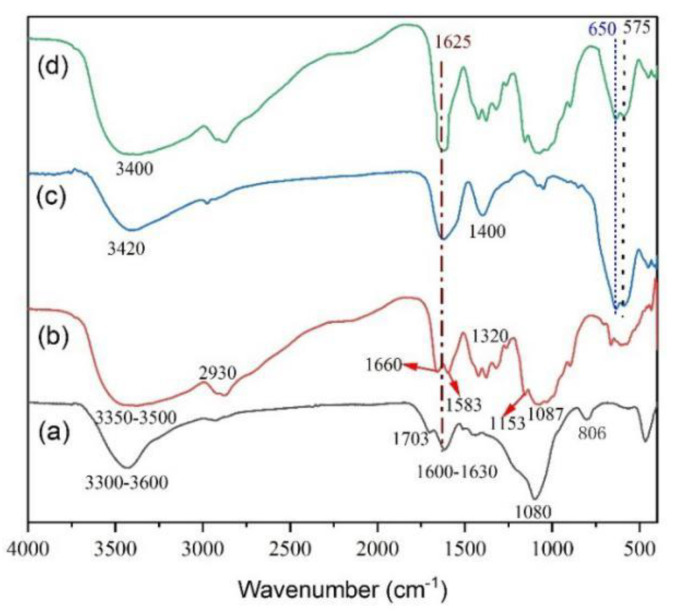
FT-IR spectra of CF (**a**), chitosan (**b**), Fe_3_O_4_ (**c**) and CFS (**d**) materials.

FT-IR spectra of CF (a), chitosan (b), Fe_3_O_4_ (c) and CFS (d) materials are shown in Figure 1. The surface of CF contained a large number of functional groups, which were beneficial for reacting with the chitosan. As can be seen from Figure 1a, the absorption band at around 3300–3600 cm^−1^ was attributed to O–H stretching vibration of the CF. The peak located at 1600 cm^−1^ was assigned to the water molecules [27]. The strong stretching vibration at 1630 cm^−1^ stands for alkene (C=C), may be related to oxygen-containing functional groups like –COOH or –OH, while the peak at 1703 cm^−1^ corresponded to the presence of carbonyl (C=O) group. It can also be observed that band at 806 cm^−1^ corresponded to –CH_2_ groups or carboxylic acid –OH groups [28]. The peak located near 1080 cm^−1^ was attributed to Si–O–Si and Si–O vibration, and it can normally be observed for CF due to its Si abundance.

From Figure 1b, the absorption band at 3350–3500 cm^−1^ was corresponded to the stretching vibrations of –OH, –NH_2_ and –NH– groups of CS material. The peaks at 1660, 1583, and 1320 cm^−1^ were attributed to the C=O, –NH– and –C–N– characteristic vibrations in the amide groups, respectively. The bands at 1153 and 1087 cm^−1^ were corresponded to the –C–O– ether groups. The band at 2930 cm^−1^ was assigned to the stretching vibration of –CH_2_ of CS material. These results indicated that –COOH and –OH groups of CF can react with –OH and –NH_2_ groups of chitosan completely. As shown in Figure 1c, the absorption peaks at 575 and 650 cm^−1^ were assigned to the stretching vibration of the Fe–O bond of Fe_3_O_4_. The broad peaks near 3420, 1625 and 1400 cm^−1^ were assigned to O–H stretching vibration and H–O–H bending vibration of adsorbed water on the Fe_3_O_4_ surface. As can be seen from Figure 1d, the broad peaks at 3400 and 1625 cm^−1^ were attributed to O–H stretching and H–O–H bending. The peaks at 575 and 650 cm^−1^ were attributed to Fe-O stretching vibrations. These results indicated that the CFS was successfully prepared.

**Figure 2:**
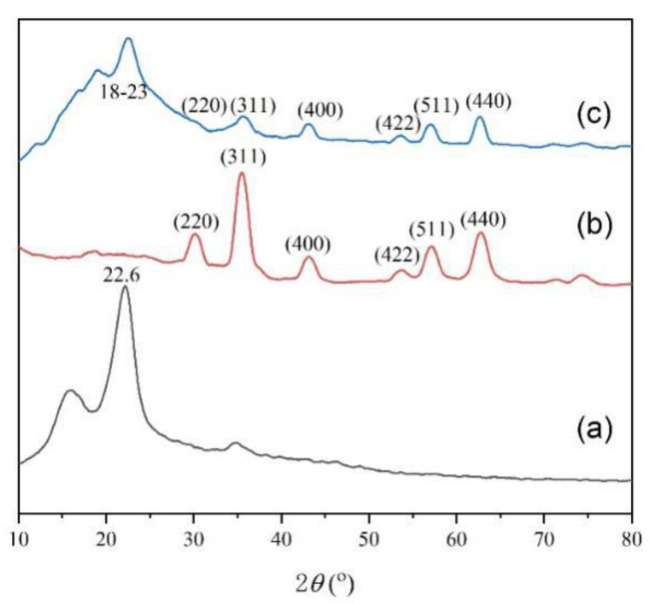
XRD patterns of CF (**a**), Fe_3_O_4_ (**b**) and CFS (**c**) materials.

**Figure 3:**
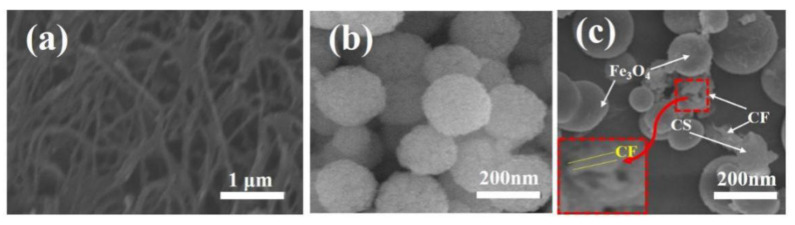
SEM images of CF (**a**), Fe_3_O_4_ (**b**) and CFS (**c**).

XRD patterns of CF (a), Fe_3_O_4_ (b) and CFS (c) materials are shown in the Figure 2. The characteristic peak at 2θ = 22.6° (Figure 2a) was attributed to carbon peak (JCPDS No. 50-0926); and its typical CF crystalline lattice peak at 15.8° was also obvious observed. From Figure 2b, the diffraction peaks at 2-theta values of 30.5°, 36.2°, 44.0°, 52.8°, 57.4° and 62.0° corresponded to the (220), (311), (400), (422), (511) and (440) phase planes of typical inverse spinel crystalline structure Fe_3_O_4_ (JCPDS No. 85-1436), respectively. As shown in Figure 2c the peak ranged from 18° to 23° should be attributed to the carbon peak of CF and amorphous state of CS. The diffraction peaks of Fe_3_O_4_ could be also observed. SEM images of the as-prepared CF (a), Fe_3_O_4_ (b) and CFS (c) are shown in the Figure 3. The width of CF was more than 0.2–0.4 µm and the surface of CF was relatively smooth (Figure 3a). From the Figure 3b, Fe_3_O_4_ core has a mean size about 150~250 nm. The CF, CS and spherical Fe_3_O_4_ were all observed in the Figure 3c. The results of SEM and XRD could be confirmed that the CFS was successfully prepared.

**Figure 4:**
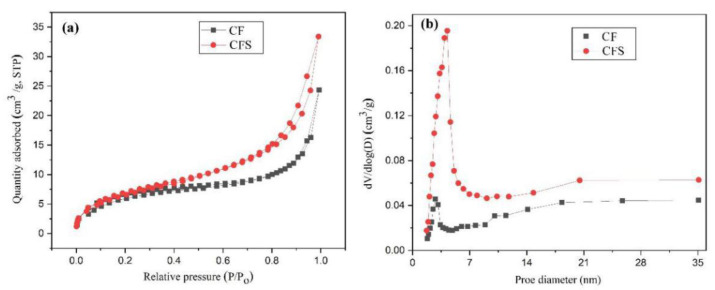
The N_2_ adsorption-desorption isotherms (**a**) and pore diameter distribution (**b**) of CF and CFS materials.

For comparation, specific surface areas of CF and CFS materials were measured. The N_2_ adsorption-desorption isotherms and pore diameter distribution of CF and CFS materials are expressed in Figure 4. The BET specific surface area (Figure 4a) of CFS had a bigger specific surface area (168.55 m^2^/g) than that of CF (120.48 m^2^/g), which could be attributed to structural advantages that the CS and Fe_3_O_4_ modified CF could bring a bigger geometrical region. This result was coincident with highly effective adsorption of CAPC by CFS adsorbent. The average pore diameters of CF and CFS materials were 2.81 and 4.26 nm, respectively (Figure 4b). Therefore, CFS material showed great CAPC removal ability from wastewater.

### 3.2. Effects of pH and Ionic Strength

**Figure 5:**
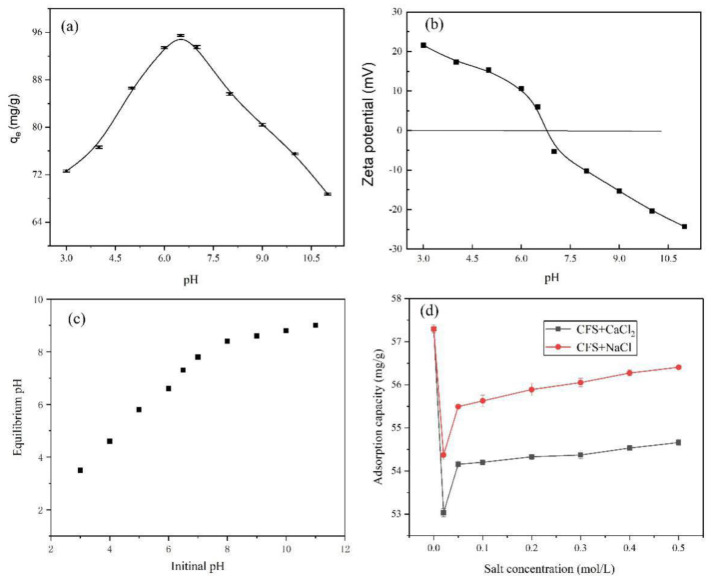
(**a**) Effect of pH on the adsorption capacity, (**b**) zeta potential of CFS adsorbent, (**c**) the recorded initial and equilibrium pH and (**d**) effect of ionic strength on the adsorption capacity (T = 308 K, t = 3 h, C_0_ = 150 mg/L, V = 20 mL, m = 30 mg).

pH value of solution is an important research parameter because it can have an effect of the charge of adsorbent and CAPC. The effects of different pH on the adsorption capacity and zeta potential of CFS adsorbent were tested by changing the pH of CAPC solutions within acid and base of proper concentration applied for adjusting the pH of solution, and the results could be found in Figure 5a,b. From Figure 5a, it was found the highest adsorption capacity observed in the pH = 6.5. This is mainly because the pH value of the solution affects the chemical valence state (ionization or neutral) of the CAPC, thus changing the adsorption characteristics of the CAPC on CFS adsorbent [29]. As can be seen from Figure 5b, accordingly, the isoelectric point (pHpzc) of CFS adsorbent was at 6.68 [10], suggesting that the surface of the prepared CFS adsorbents were positively charged at pH < 6.68 and the negatively charged at pH > 6.68. At low pH, the CAPC molecule was better non-ionized. The electrostatic interaction and H-bond interaction between the CAPC molecule and the O-containing functional groups of CFS adsorbent were enhanced, thus resulting in a high adsorption phenomenon. Meanwhile, after adsorption equilibrium, final (equilibrium) solution pH (pH_eq_) was also determined, and results are shown in Figure 5c. In the range of initial pH 3–6.5, the pH_eq_ strongly increases: protons are bound to adsorbent matrix. In the range of initial pH 7–11, the pH_eq_ tends to stabilize around 7.8–9.0. In addition, the leaching degree of iron in the solutions of different pH was tested by the method of inductively coupled plasma-optical emission spectrometry (ICP-OES, VISTA-MPX, Varian Ltd., Victoraia, Australia) [14], and the stability of CFS adsorbent was explored. The results showed that the content of iron dissolved in aqueous solution was less than 0.102 mg/L after adsorption, showing that the prepared CFS adsorbent had good stability. Based on the above analysis obtained results, the most suitable optimum pH in the process of adsorption CPAC removal on CFS was 6.5.

In this work, NaCl and CaCl_2_, two common salts, were used to judge the effects of ionic strength on the process of CAPC adsorption on CFS. As shown in Figure 5d, the Ca^2+^ showed a stronger inhibition impact on the adsorption capacity than the Na^+^. With the increase in salt concentration from 0 to 0.02 mol/L, the adsorption capacity decreased initially. This may be because the shielding effect of surface charge at low salt concentration inhibits the intermolecular attraction between CAPC and the surface of CFS, leading to the decrease in adsorption capacity [30]. When the concentration increasing from 0.02 to 0.05 mol/L, the adsorption capacity augmented, and when the concentration aggrandized to 0.5 mol/L, the adsorption capacity changed little. This may be mainly because the effect of salting out would reduce the solubility of CAPC in aqueous solution with the increasing of ionic strength. The decrease in solubility promoted the diffusion of more CAPC molecules to the adsorbent surface, thus improving the adsorption capacity [31]. It could also be seen from the Figure 5d, although the adsorption capacity was reduced, the reduction caused was only 7.4%. Therefore, the obtained results showed that the prepared CFS adsorbent had potential application prospects in the treatment of wastewater containing CAPC.

### 3.3. Adsorption Isotherm Model

The experimental data for the adsorption of CAPC onto CFS adsorbent were fitted to LIM, FIM, TIM and DRIM. The linear fitting curves of LIM (a), effect of initial concentration of CAPC and temperature on the separation factor *R_L_* of LIM (b), the linear fitting curves of FIM (c), TIM (d) and DRIM (e) are shown in the Figure S2. The detailed fitting parameters and correlation coefficients of each isotherm model are listed in Table 1.

From Table 1 and Figure S2a, the linear correlation coefficient (*R*^2^) of LIM was in the range of 0.9916–0.9990, and the fitting curves was basically consistent with the experimental plots. Simultaneously, sum of squared residual error (SSRE) of LIM was much smaller than the other three models. From the Figure S2c–e), the linear correlation coefficients (*R*^2^) of FIM, TIM and DRIM were in the range of 0.9369–0.9837, 0.7273–0.8338 and 0.7988–0.8618, respectively, and the fitting curves were inconsistent with the experimental data. The correlation coefficients correlations of the isotherms were in the order: LIM > FIM > DRIM > TIM. These results indicated that the LIM was more appropriate to fit the experimental data than the other three (FIM, TIM and DRIM) models. Thus, the monomolecular layer adsorption could play a significant role, and the maximum adsorption capacities were 53.996, 58.7544, 50.1756 mg/g at temperatures of 298, 308 and 318 K, respectively. The properties of other previously reported CAPC are listed in Table 2. Meanwhile, K_L_ value increased, which may be related to the increase in temperature and the inherent chemical properties of the adsorbent. From Figure S2b, the separation factor R_L_ changed from 0.068 to 0.498, 0.068 to 0.355 and 0.058 to 0.316 at 298, 308 and 318 K, respectively. The result indicated that the adsorption step was beneficial in the specific concentration range [32]. In the case of the FIM, the constant of Freundlich adsorption (K_f_) increased with increasing adsorption temperatures. High values of K_f_ indicated that there was very good interaction between CFS and CAPC.

### 3.4. Kinetics Models

The adsorption kinetics of CFS adsorbent with initial CAPC concentration of 150 mg/L was investigated under the optimal adsorption conditions. The results of PFO (a), PSO (b) and IDK (c) models are shown in Figure S3. The kinetic parameters obtained from different kinetic models are summarized in Table 3. In Table 3, the PSO model fitted the results better compared to PFO and IDK models, and well described the kinetic model of the process (*R*^2^ = 0.9999), indicating that the adsorption of CAPC onto CFS was controlled mainly by chemisorption process [36]. Simultaneously, SSRE of PSO was much smaller than the other two models. The IDK curves were treated to test whether internal particle diffusion or external diffusion was the rate-limiting step. The IDK plot did not match the origin of coordinates (Figure S3c), and the curve showed a spinodal [10]. This phenomenon showed that the adsorption mechanism was influenced by multi-mechanisms, and the internal particle diffusion may be not the main element [37]. Therefore, external mass transfer only played a significant role in the previous part of adsorption.

### 3.5. Thermodynami

Thermodynamic Study

The thermodynamics of adsorption experiments of CFS adsorbent were conducted at 298, 308 and 318 K, at the original CPAC concentration of 150 mg/L. *K_c_* (thermodynamic equilibrium constant) was calculated using Equation (11).

(11) $$K_{c}=\frac{C_{o}-C_{e}}{C_{e}\;}=\frac{q_{e}M}{C_{e}V\;}$$

where *C*_0_ and *C_e_* are defined as the initial concentration and adsorption equilibrium concentration (mg/L), respectively, *M* is the weight of the adsorbent (g), and *V* is the volume of the mixture (mL).

Based on Van’t Hoff equation, it could be shown as the following Equations (12)–(14).

(12) $$\Delta G^{o}=-RT\ln K_{c}$$

(13) $$\Delta G^{o}=\Delta H^{o}-T\Delta S^{o}$$

(14) $$\;\ln K_{c}=-\frac{\Delta H^{o}}{RT}+\frac{\Delta S^{o}}{R}$$

where Δ*H^o^* (kJ/mol) and Δ*S^o^* (J/mol^.^K) are treated as the change of enthalpy and entropy, individually. Δ*G^o^* (kJ/mol) was defined as the Gibbs free energy. *R* is the gas constant (8.314 J/(mol^.^K)). *T* is the absolute temperature (K).

Based on the slope and intercept of a linear regression equation with one variable, Δ*H^o^* = 26.31 kJ/mol and Δ*S^o^* = 109.69 J/mol^.^K. The results suggested that the adsorption step of CAPC is an endothermic reaction and increasing temperature is benefit for fortifying the efficiency of adsorption. The positive value of Δ*S^o^* revealed the entropy-increasing nature and the increase in the randomness at the solid–liquid interface during the CAPC adsorption on CFS. The ΔG^o^ values (298 K: −6.37 kJ/mol; 308 K: −7.47 kJ/mol; 318 K: −8.57 kJ/mol) were negative, which showed that process of CAPC adsorption on CFS was a spontaneous physisorption step. Above results indicated that CFS could be used for highly efficient CAPC removal from wastewater.

### 3.6. Recycling Ability and Adsorbent Mechanism

**Figure 6:**
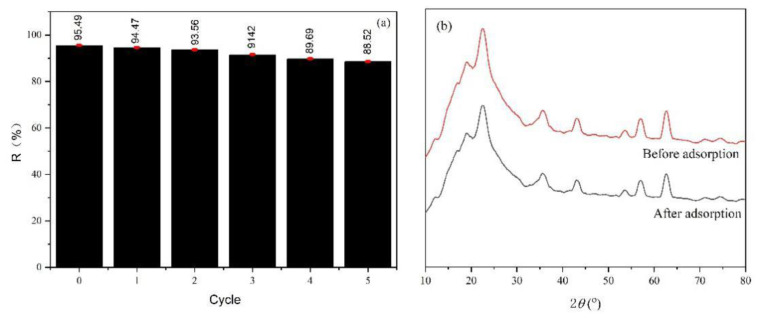
Reusability of CFS for CAPC adsorption (**a**) and XRD patterns (**b**) before and after adsorption of CFS adsorbent (Adsorption time = 3 h, C_0_ = 150 mg/L, V = 20 mL, m = 30 mg, pH = 6.5).

To determine the recycling ability of the adsorbent, we investigated the repetition of the CAPC adsorption-desorption cycle. The results of this study are demonstrated in Figure 6a. The CAPC removal rate was approximately 95.49% after the 1st cycle and decreased to 88.52% after 5th cycles. The CFS material showed excellent recycling ability. Moreover, the magnetic part is in favor of separation step conveniently. Figure 6b showed that there was little change in XRD patterns before and after use, indicating that CFS had excellent stability after use. So, the CFS adsorbent may be an outstanding potential candidate in wastewater treatment.

CAPC is a kind of amide compound, containing p-nitro (–NO_2_), hydroxyl (–OH), amine (–NH) and carbonyl (C=O) active groups, which can form hydrogen bonds with -hydroxyl, carboxyl and amino groups. There are a great number of –OH groups on the surface of corn stalk biomass (CF) fiber O–H, which can combine with CAPC by hydrogen bond. The chitosan possesses hydroxyl (–OH) and amino (–NH_2_), which can react with CAPC. From the comparison, CFS presented in this study showed superior adsorption capacity compared to the other materials, highlighting its potential for CPAC removal in wastewater.

## 4. Conclusions

In conclusion, a novel adsorbent (CFS) using corn stalk biomass fiber and Fe_3_O_4_ embedded chitosan was prepared and applied for removal of chloramphenicol (CAPC). The adsorption experiments results showed the CFS had excellent adsorption ability and good recycling performance. The adsorption equilibrium data are in agreement with the Langmuir model better and best-fit the pseudo-second order kinetics. The maximum adsorption capacity of CFS (from the Langmuir model) was obtained 58.75 mg/g. This study reports a novel adsorbent as a promising candidate for the specific adsorption of CPAC-containing substances.

## Figures and Tables

**Table 1:** Comparison of LIM, FIM, TIM and DRIM isotherm parameters obtained for CAPC-CFS adsorption system (t = 3 h, V = 20 mL, m = 30 mg, pH = 6.5).

Model	Parameter	298 K	308 K	318 K
LIM	*q_e, cal_* (mg/g)	59.3600	62.7933	63.1200
	*q_m_* (mg/g)	53.996	58.7544	50.1756
	*K_L_* (L/mg)	0.0504	0.0909	0.1084
	*R* ^2^	0.9916	0.9990	0.9958
	*SSRE* (×10^−5^)	3.9191	0.3743	1.5981
FIM	*K_f_* (mg^1−(1/n)^ L^1/n ^ g^−1^)	1.4007	4.1343	4.2137
	1/*n*	1.61553	1.5744	1.6628
	*R* ^2^	0.9369	0.9837	0.9677
	*SSRE*	0.2042	0.0499	0.0994
TIM	*b_T_* (J^.^g/mol^.^mg)	44.6432	43.1616	42.4106
	*K_T_* (L/mg)	0.3015	0.53889	0.56649
	*R* ^2^	0.7273	0.8338	0.8029
	*SSRE* (×10^3^)	1.3410	0.8555	1.0274
DRIM	*q_m_* (mg/g)	71.9111	81.6123	85.8429
	*K_DR_* (molJ)	4.8 × 10^−6^	1.8 × 10^−6^	1.7 × 10^−6^
	*R* ^2^	0.7988	0.8618	0.8612
	*SSRE*	0.6508	0.4246	0.4270

**Table 2:** Previous studies on the adsorption of CAPC with different adsorbents.

Adsorbents	Temperature (K)	*q_e_* (mg/g)	Ref.
Si@MIPs-CAP	298	32.258	[3]
BC (Bamboo charcoal)	-	8.1	[33]
FBC	298	21.35	[34]
MRBC	298	9.29	[14]
RBC	298	6.14	[14]
Fe_3_O_4_@mSiO_2_@NIP	298	44	[35]
Aligned-MWCNT	298	14.58	[29]
CFS	298–308	50.18–58.75	This work

**Table 3:** Comparison of PFO, PSO and IDK kinetic parameters obtained for CAPC-CFS adsorption system (C_0_ = 150 mg/L, V = 20 mL, m = 30 mg, pH = 6.5).

Model	Parameter	298 K	308 K	318 K
PFO	*q_e_* (mg/g)	38.5837	38.7306	38.9391
	*K*_1_ (/min)	0.0013	0.0011	0.0011
	*R* ^2^	0.5687	0.5220	0.5236
	*SSRE*	0.0699	0.0664	0.0638
PSO	*q_e_* (mg/g)	33.0142	42.3012	41.0846
	*K*_2_×10^−3^ (g/(mg min))	8.5363	10.2812	9.9088
	*R* ^2^	0.9999	0.9999	0.9999
	*SSRE* (×10^−4^)	3.3791	0.7734	1.7798
IDK	*K_p_*_1_ (mg/g/min^1/2^)	2.4282	2.3006	2.2739
	*C*_1_ (mg/g)	43.3067	46.6520	47.0841
	*R* ^2^	0.9305	0. 8621	0.8519
	*SSRE*	8.9674	0.0469	17.2486
	*K_p_*_2_ (mg/g/min^1/2^)	0.1209	0.0921	0.1197
	*C*_2_ (mg/g)	59.8534	62.3396	62.1838
	*R* ^2^	0.9125	0.9519	0.8625
	*SSRE*	0.0121	18.2991	0.0765

## Data Availability

The data presented in this study are available on request from the corresponding author.

## Supplementary Materials

The following are available online at https://www.mdpi.com/article/10.3390/nano11112950/s1, Figure S1: The molecular structure of chloramphenicol and corn stalk wastes; Figure S2: The linear fitting of Langmuir (a), effect of initial concentration of CAPC and temperature on the separation factor R_L_ of LIM (b), the linear fitting curves of FIM (c), TIM (d) and DRIM (e) t = 3 h, V = 20 mL, m = 30 mg, pH = 6.5); Figure S3: PFO (a), PSO (b) and IDK (c) adsorption kinetic curves (C_0_ = 150 mg/L, V = 20 mL, m = 30 mg, pH = 6.5).

## References

[1] Ahammad N.A., Zulkifli M.A., Ahmad M.A., Hameed B.H., Mohd Din A.T. (2021). Desorption of chloramphenicol from ordered mesoporous carbon-alginate beads: Effects of operating parameters, and isotherm, kinetics, and regeneration studies. J. Environ. Chem. Eng..

[2] Rahimi Z., Shahbazi Y., Ahmadi F. (2016). Polypyrrole as an efficient solid-phase extraction sorbent for determination of chloramphenicol residue in chicken liver, kidney, and meat. Food Anal. Methods.

[3] Yang F., Zhang Q., Jian H.X., Wang C.P., Xing B.S., Sun H.W., Hao Y.L. (2020). Effect of biochar-derived dissolved organic matter on adsorption of sulfamethoxazole and chloramphenicol. J. Hazard. Mater..

[4] Dong H., Qiang Z., Hu J., Qu J. (2017). Degradation of chloramphenicol by UV/chlorine treatment: Kinetics, mechanism and enhanced formation of halonitromethanes. Water Res..

[5] Xu H., Zhang Y., Li J., Hao Q., Li X., Liu F. (2020). Heterogeneous activation of peroxymonosulfate by a biochar-supported Co_3_O_4_ composite for efficient degradation of chloramphenicols. Environ. Pollut..

[6] Han X., Li P., Zhang M., Wang J., Gao Y., Zhang T., Zhou G., Li F. (2021). Designing three-dimensional half-embedded ES-PAN/AHCNs adsorption membrane for removal of Pb(II), Cu(II) and Cr(III). Colloids Surf. A.

[7] Ighalo J.O., Adeniyi A.G., Adelodun A.A. (2021). Recent advances on the adsorption of herbicides and pesticides from polluted waters: Performance evaluation via physical attributes. J. Ind. Eng. Chem..

[8] Wu G.Y., Liu Q., Wang J.Y., Xia S.Y., Wu H.L., Zong J.X., Han J.G., Xing W.N. (2021). Facile fabrication of rape straw biomass fiber/β-CD/Fe_3_O_4_ as adsorbent for effective removal of ibuprofen. Ind. Crop. Prod..

[9] Mukwevho N., Gusain R., Fosso-Kankeu E., Kumar N., Waanders F., Ray S.S. (2020). Removal of naphthalene from simulated wastewater through adsorption-photodegradation by ZnO/Ag/GO nanocomposite. J. Ind. Eng. Chem..

[10] Yuan J., Zhu Y., Wang J., Gan L., He M., Zhang T., Li P., Qiu F. (2021). Preparation and application of Mg-Al composite oxide/coconut shell carbon fiber for effective removal of phosphorus from domestic sewage. Food Bioprod. Process.

[11] Liu H., Li P., Yu H., Zhang T., Qiu F. (2019). Controlled fabrication of functionalized nanoscale zero-valent iron/celluloses composite with silicon as protective layer for arsenic removal. Chem. Eng. Res. Des..

[12] Zhu W., Jiang X., Jiang K., Liu F., You F., Yao C. (2021). Fabrication of reusable carboxymethyl cellulose/graphene oxide composite aerogel with large surface area for adsorption of methylene blue. Nanomaterials.

[13] Ahmed M.B., Zhou J.L., Ngo H.H., Guo W., Johir M.A.H., Sornalingam K., Belhaj D., Kallel M. (2017). Nano-Fe_0_ immobilized onto functionalized biochar gaining excellent stability during sorption and reduction of chloramphenicol via transforming to reusable magnetic composite. Chem. Eng. J..

[14] Zhao H., Lang Y. (2018). Adsorption behaviors and mechanisms of florfenicol by magnetic functionalized biochar and reed biochar. J. Taiwan Inst. Chem. Eng..

[15] Elwakeel K.Z., Elgarahy A.M., Khan Z.A., Almughamisi M.S., Al-Bogami A.S. (2020). Perspectives regarding metal/mineral-incorporating materials for water purification: With special focus on Cr(VI) removal. Mater. Adv..

[16] Elwakeel K.Z., Al-Bogami A.S., Elgarahy A.M. (2018). Efficient Retention of chromate from industrial wastewater onto a green magnetic polymer based on shrimp peels. J. Polym. Environ..

[17] Elwakeel K.Z., El-Bindary A.A., Ismail A., Morshidy A.M. (2017). Magnetic chitosan grafted with polymerized thiourea for remazol brilliant blue R recovery: Effects of uptake conditions. J. Dispers. Sci. Technol..

[18] Elwakeel K.Z., Atia A.A., Guibal E. (2014). Fast removal of uranium from aqueous solutions using tetraethylenepentamine modified magnetic chitosan resin. Bioresour. Technol..

[19] Zhu Y., Rong J., Mao K., Yang D., Zhang T., Qiu F., Pan J. (2020). Fe_3_O_4_@chitosan-bound boric acid composite as pH-responsive reusable adsorbent for selective recognition and capture of cis-diol-containing shikimic acid. Appl. Organomet. Chem..

[20] Wang J., Yang F. (2021). Preparation of 2-hydroxypropyl-b-cyclodextrin polymers crosslinked by poly(acrylic acid) for efficient removal of chloramphenicol. Mater. Lett..

[21] Langmuir I. (1916). The constitution and fundamental properties of solids and liquids. Part I. Solids. J. Am. Chem. Soc..

[22] Yuan J., Qiu F., Li P. (2017). Synthesis and characterization of β-cyclodextrin-carboxymethyl cellulose-graphene oxide composite materials and its application for removal of basic fuchsin. J. Iran. Chem. Soc..

[23] Weber T.W., Chakravorti R.K. (1974). Pore and solid diffusion models for fixed-bed adsorbers. J. Am. Inst. Chem. Eng..

[24] Yang Y., Chen N., Feng C., Li M., Gao Y. (2018). Chromium removal using a magnetic corncob biochar/polypyrrole composite by adsorption combined with reduction: Reaction pathway and contribution degree. Colloids Surf. A.

[25] Kong J., Zheng Y., Xiao L., Dai B., Meng Y., Ma Z., Wang J., Huang X. (2020). Synthesis and comparison studies of activated carbons based folium cycas for ciprofloxacin adsorption. Colloids Surf. A.

[26] Shi Z., Xu C., Guan H., Li L., Fan L., Wang Y., Liu L., Meng Q., Zhang R. (2018). Magnetic metal organic frameworks (MOFs) composite for removal of lead and malachite green in wastewater. Colloids Surf. A.

[27] Ogungbenro A.E., Quang D.V., Al-Ali K.A., Vega L.F., Abu-Zahr M.R.M. (2020). Synthesis and characterization of activated carbon from biomass date seeds for carbon dioxide adsorption. J. Environ. Chem. Eng..

[28] Li M., Zhang Z., Li Z., Wu H. (2020). Removal of nitrogen and phosphorus pollutants from water by FeCl_3_-impregnated biochar. Ecol. Eng..

[29] Zhao H., Liu X., Cao Z., Zhan Y., Shi X., Yang Y., Zhou J., Xu J. (2016). Adsorption behavior and mechanism of chloramphenicols, sulfonamides, and non-antibiotic pharmaceuticals on multi-walled carbon nanotubes. J. Hazard. Mater..

[30] Fröhlich A.C., dos Reis G.S., Pavan F.A., Lima É.C., Foletto E.L., Dotto G.L. (2018). Improvement of activated carbon characteristics by sonication and its application for pharmaceutical contaminant adsorption. Environ. Sci. Pollut. Res..

[31] Peng X., Hu F., Lam F., Wang Y., Liu Z., Dai H. (2015). Adsorption behavior and mechanisms of ciprofloxacin from aqueous solution by ordered mesoporous carbon and bamboo-based carbon. J. Colloid Interface Sci..

[32] Martins A.C., Pezoti O., Cazetta A.L., Bedin K.C., Yamazaki D.A.S., Bandoch G.F.G., Asefa T., Visentainer J.V., Almeida V.C. (2015). Removal of tetracycline by NaOH-activated carbon produced from macadamia nut shells: Kinetic and equilibrium studies. Chem. Eng. J..

[33] Liao P., Zhan Z., Dai J., Wu X., Zhang W., Wang K., Yuan S. (2013). Adsorption of tetracycline and chloramphenicol in aqueous solutions by bamboo charcoal: A batch andfixed-bed column study. Chem. Eng. J..

[34] Ahmed M.B., Zhou J.L., Ngo H.H., Guo W., Johir M.A.H., Belhaj D. (2017). Competitive sorption affinity of sulfonamides and chloramphenicol antibiotics toward functionalized biochar for water and wastewater treatment. Bioresour. Technol..

[35] Wei S., Li J., Liu Y., Ma J. (2016). Development of magnetic molecularly imprinted polymers with double templates for the rapid and selective determination of amphenicol antibiotics in water, blood, and egg samples. J. Chromatogr. A.

[36] Bedin K.C., Martins A.C., Cazetta A.L., Pezoti O., Almeida V.C. (2016). KOH-activated carbon prepared from sucrose spherical carbon: Adsorption equilibrium, kinetic and thermodynamic studies for methylene blue removal. Chem. Eng. J..

[37] Drweesh S.A., Fathy N.A., Wahba M.A., Hanna A.A., Akarish A.I.M., Elzahany E.A.M., El-Sherif I.Y., Abou-El-Sherbini K.S. (2016). Equilibrium, kinetic and thermodynamic studies of Pb(II) adsorption from aqueous solutions on HCl-treated Egyptian kaolin. J. Environ. Chem. Eng..

