# Supplementary material for: High Selectivity and Reusability of Biomass-Based Adsorbent for Chloramphenicol Removal
Source: Nanomaterials (Basel). 2021 Nov 3;11(11):2950. doi: 10.3390/nano11112950 (PMC8621775; doi:10.3390/nano11112950)
Supplement: Supplementary file 1 [file nanomaterials-11-02950-s001.zip › nanomaterials-1435096-supplementary.pdf]

High Selectivity and Reusability of Biomass-Based Adsorbent for Chloramphenicol Removal

Weinan Xing [1,2,*], Qi Liu [1], Jingyi Wang [1], Siye Xia [1], Li Ma [1], Ran Lu [1], Yujing Zhang [1], Yudong Huang [3] and Guangyu Wu [1,2,4,*]

[1] Co-Innovation Center for the Sustainable Forestry in Southern China, College of Biology and the Environment, Nanjing Forestry University, Nanjing 210037, China; liuqichem@126.com (Q.L.); wangjychem@126.com (J.W.); siyexia2021@126.com (S.X.); limanjfu@163.com (L.M.); luranNJLR@126.com (R.L.); yjzhang2018@126.com (Y.Z.)

[2] National Positioning Observation Station of Hung-tse Lake Wetland Ecosystem in Jiangsu Province, Hongze 223100, China

[3] MIIT Key Laboratory of Critical Materials Technology for New Energy Conversion and Storage, State Key Laboratory of Urban Water Resource and Environment, School of Chemistry and Chemical Engineering, Harbin Institute of Technology, Harbin 150001, China; huangyd@hit.edu.cn

[4] Jiangsu Key Laboratory of Advanced Catalytic Materials and Technology, Changzhou University, Changzhou 213164, China

* Correspondence: xingwn@njfu.edu.cn (W.X.); gywuchem@ njfu.edu.cn (G.W.)

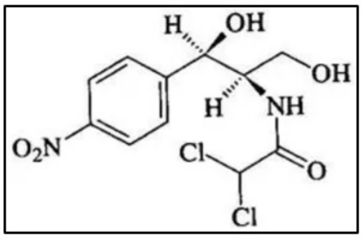

(a)

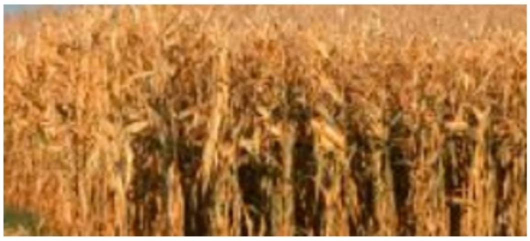

(b)

Figure S1. The molecular structure of chloramphenicol (a) and corn stalk wastes (b).

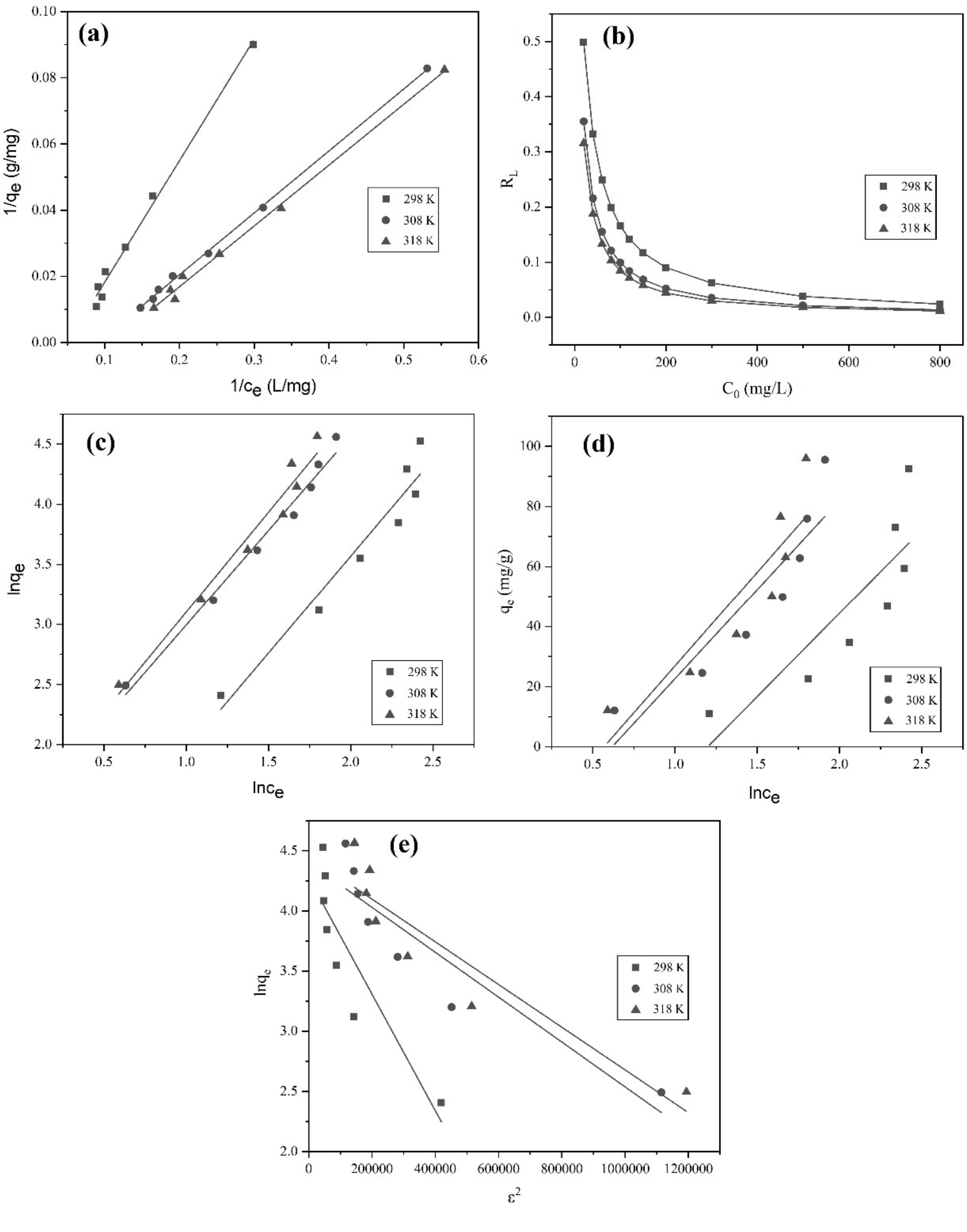

Figure S2. The linear fitting of Langmuir (a), effect of initial concentration of CAPC and temperature on the separation factor R_L of LIM (b), the linear fitting curves of FIM (c),TIM (d) and DRIM (e) (t =3 h, V= 20 mL, m =30 mg, pH=6.5).

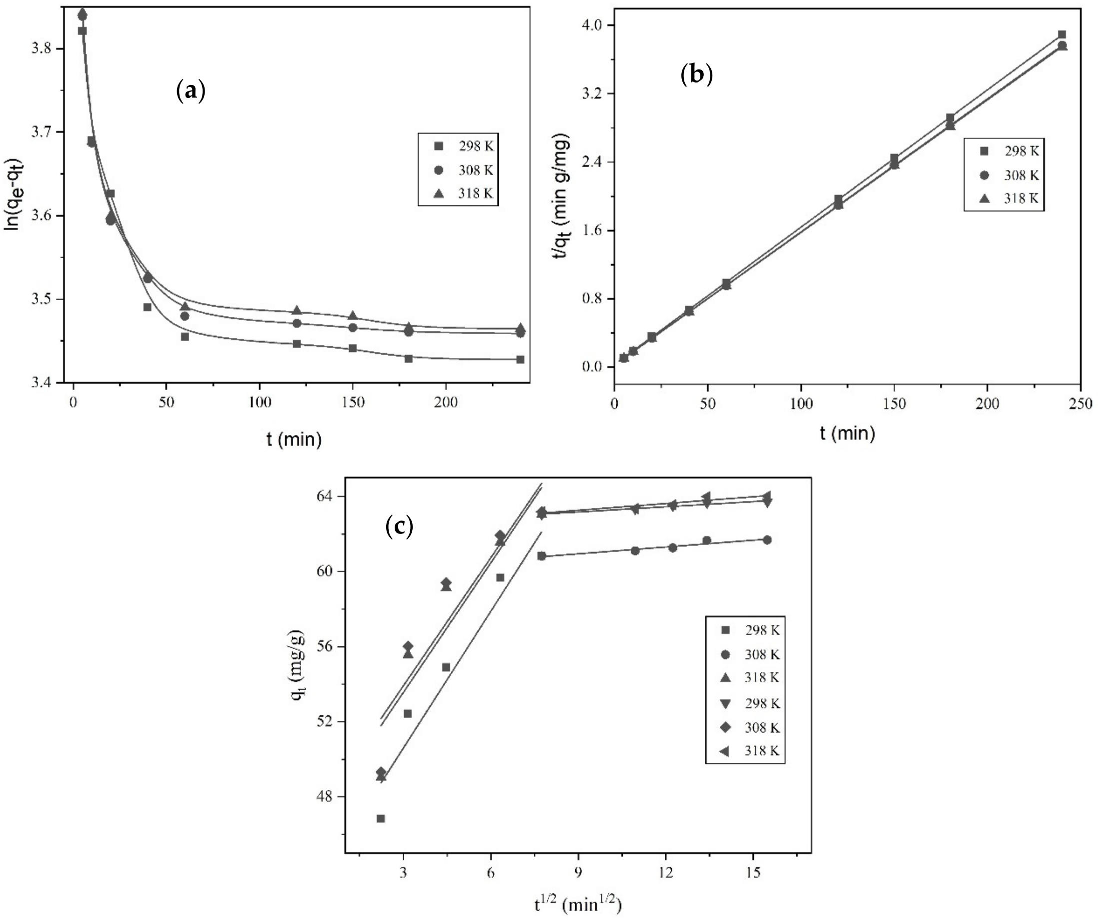

Figure S3. PFO (a), PSO (b) and IDK (c) adsorption kinetic curves (C_0=150 mg/L, V= 20 mL, m =30 mg, pH=6.5).
